# SMG-Net: A lightweight modular architecture for fine-grained crack segmentation in ancient wooden structures

**DOI:** 10.1371/journal.pone.0336125

**Published:** 2025-11-19

**Authors:** Tianke Fang, Zhenxing Hui, Zhiying Xie, Peng Yu, Yi Gao, Songdi Shi, Yuanrong He

**Affiliations:** 1 School of Computing and Information Engineering, Xiamen University of Technology, Xiamen, Fujian, China; 2 Big Data Institute of Digital Natural Disaster Monitoring in Fujian, Xiamen University of Technology, Xiamen, Fujian, China; 3 Faculty of Innovation Engineering, Macau University of Science and Technology, Taipa, Macau, China; 4 School of Software, Liaoning Technical University, Huludao, Liaoning, China; Islamic Azad University Mashhad Branch, IRAN, ISLAMIC REPUBLIC OF

## Abstract

To improve the accuracy and efficiency of crack segmentation in ancient wooden structures, we propose a lightweight deep neural network architecture, termed SMG-Net. The core innovation of this model lies in its multi-cooperative perception mechanism. First, the proposed Structure-Aware Cross-directional Pooling (SACP) establishes long-range feature dependencies in multiple orientations, addressing the challenge of coherent recognition for cracks with complex directions. Second, the Multi-path Robust Feature Extraction (MRFE) module enhances the tolerance of the model to noise and blurred edges, thereby improving the discriminative capability of shallow features. Third, the Guided Semantic–Spatial Fusion (GSSFusion) mechanism enables efficient alignment and integration of multi-scale features, ensuring both fine crack details and global structural consistency in segmentation. Extensive experiments were conducted on a self-constructed dataset of cracks in ancient wooden components and the public Masonry crack dataset. SMG-Net achieved mean Intersection-over-Union (mIoU) scores of 81.12% and 87.91%, and Pixel Accuracy (PA) of 98.91% and 98.99%, respectively, significantly outperforming mainstream approaches such as U-Net, SegFormer, and Swin-UNet, with results confirmed by statistical significance testing. Moreover, SMG-Net demonstrates superior parameter efficiency and inference speed, making it particularly suitable for heritage monitoring scenarios with limited computational resources. To promote reproducibility and future research, the source code and datasets have been made publicly available at: https://github.com/HuiZhenxing/HuiZhenxing.git.

## Introduction

The timber architecture of the Minnan region has a long history and holds significant cultural value. Over time, natural environmental factors have caused varying degrees of deterioration in these historic buildings, with cracks in wooden components being the most common issue, posing serious threats to both structural safety and cultural heritage preservation [1]. Traditional crack inspection methods rely heavily on manual observation, which is labor-intensive, time-consuming, and prone to subjective bias, limiting their scalability and precision. Recent advances in computer vision and deep learning offer promising alternatives, with image semantic segmentation enabling pixel-level classification for precise crack localization and measurement [2].

Among segmentation approaches, convolutional neural network (CNN)-based methods have become dominant in crack detection [3–5]. CNNs are effective at extracting local features, and numerous architectures have been developed to handle thin, elongated, and irregularly shaped cracks. Long et al. [6] introduced the fully convolutional network (FCN), initiating deep learning applications in image segmentation. Subsequent improvements combining CNN backbones with FCN aimed to enhance segmentation accuracy and generalization. Feature Pyramid Networks (FPN) were proposed to incorporate multi-scale features, improving spatial detail and semantic representation; however, their lateral connections are limited in capturing long-range dependencies, restricting the ability to model global crack structures [7].

U-Net [8] further addressed context modeling limitations by employing a symmetric encoder-decoder architecture with skip connections, effectively preserving spatial details while maintaining high-level semantic features. U-Net and its variants have shown strong performance in crack segmentation [9–11], but challenges remain in complex building scenes, where cracks exhibit diverse orientations, subtle features, and uneven distributions.

To overcome these limitations, several enhanced architectures have been explored. VM-UNet [12] and H-vmUNet [13] integrate state-space models with contextual attention for improved long-range dependency modeling. CNN-based improvements, such as ResNet encoders with ASPP [14], SegNet [15], and PSPNet with spatial attention [16,17], improve boundary recognition but struggle with small-scale cracks in complex backgrounds. Attention-based networks including AttU-Net [18], ABCNet [19], CMUNet [20], ENet [21], and A2-FPN [22] enhance local feature extraction, yet global structural perception remains limited.

Transformer-based methods, with strong global modeling capabilities, have recently been applied to image segmentation, often in combination with CNNs to integrate local and global features [23–30]. SegFormer [31] and Swin-Unet [32] demonstrate superior multi-scale context modeling and fine structural recognition, though model complexity and training cost hinder lightweight deployment. MALUNet [33] combines multi-scale information with attention mechanisms to maintain efficiency and improve segmentation accuracy, yet it still faces limitations with complex crack orientations and uneven image responses.

In summary, although existing methods have made progress in crack segmentation accuracy, lightweight design, and adaptability to complex backgrounds, they remain challenged by the characteristics of timber cracks in historic buildings, such as variable orientations, subtle features, uneven distributions, and multi-scale feature fusion limitations. To address these challenges, this study proposes a structure-enhanced pixel-level crack segmentation network, SMG-Net, with the following innovations:

1. **Structure-aware Cross-directional Pooling (SACP)**: Models strip-like contextual information along horizontal, vertical, and diagonal directions, enhancing the network’s sensitivity to cracks with multiple orientations;
2. **Multi-path Robust Feature Extraction (MRFE)**: Integrates convolution, slice-reorganization, gating mechanisms, and diverse pooling strategies to strengthen shallow feature representation and reduce detail loss caused by downsampling;
3. **Guided Semantic-Spatial Fusion (GSSFusion)**: Combines channel attention with spatial guidance to coordinate feature fusion across different levels, improving perception and localization in non-uniform crack regions.

The collaborative effect of these modules enables SMG-Net to effectively handle cracks with complex morphologies, strong background interference, and blurred texture boundaries. Experimental results demonstrate that SMG-Net outperforms existing mainstream methods across multiple evaluation metrics, exhibiting strong generalization ability and practical application potential.

## Materials and methods

### Overall structure of SMG-Net

**Fig 1:**
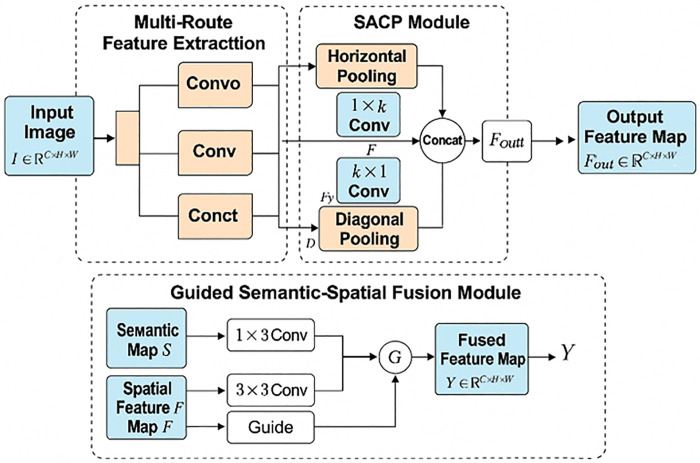
SMG-Net network structure.

To address the challenges of segmenting cracks in wooden architectural components—such as variable structural orientations, weak fine-grained features, and complex background scenes—this study proposes a Structure-Enhanced Pixel-Level Crack Segmentation Network (SMG-Net). The overall architecture of SMG-Net is illustrated in Fig 1. The network integrates multiple attention mechanisms with lightweight modules to enhance multi-scale contextual modeling and fine structural representation, while maintaining low computational cost and parameter count, making it suitable for practical deployment.

SMG-Net is built upon an improved U-Net backbone, where depthwise separable convolutions replace standard convolutions. This substitution reduces computational complexity and the number of parameters while preserving feature representation capability, improving the network’s efficiency and suitability for lightweight applications.

**Fig 2:**
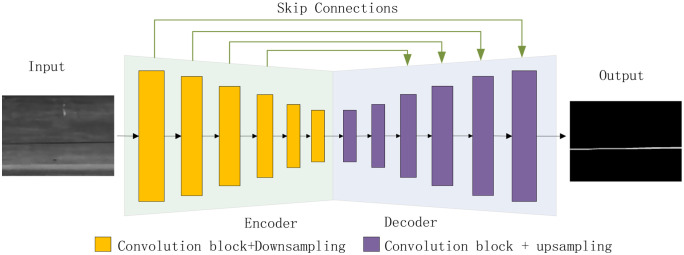
Schematic diagram of encoder-decoder architecture.

The network employs a six-level encoder with progressively increasing channel numbers to deepen feature extraction. The encoder follows the typical contraction path of image segmentation networks, replacing any fully connected layers with convolutional layers. A symmetric decoder is appended on top of the encoder, comprising consecutive convolutional layers followed by upsampling layers. The encoder captures contextual information, whereas the decoder achieves precise localization. Skip connections enable the decoder to access low-level features from the encoder, mitigating information loss. The encoder-decoder structure is depicted in Fig 2.

To address the efficiency limitations of conventional multi-head self-attention mechanisms in small-sample semantic segmentation, as well as the constraints of U-Net skip connections in maintaining semantic consistency and contextual fusion, we propose the Structure-Aware Cross-directional Pooling (SACP) module. This module effectively captures elongated cracks, diagonal textures, and local structural information within the image, enhancing the network’s structural sensitivity and spatial dependency modeling without introducing additional computational overhead.

During feature extraction and downsampling, SMG-Net incorporates the Multi-Route Feature Extractor (MRFE) module. By employing a multi-path feature extraction strategy, this module processes input images in parallel to capture semantic and texture information from multiple perspectives, thereby improving the model’s robustness to small-scale cracks and complex backgrounds.

To achieve precise crack boundary recovery and detailed feature perception, SMG-Net further integrates the Guided Semantic-Spatial Fusion (GSSFusion) module. This module combines semantic guidance and spatial positional guidance mechanisms to selectively fuse feature maps from different scales and semantic levels, enhancing the perception of boundary regions and improving the detection accuracy for subtle crack structures.

Finally, the network outputs are restored to the original resolution using bilinear interpolation, and a Sigmoid activation function is applied to generate binary segmentation results for pixel-level crack detection. The overall network architecture maintains high segmentation accuracy while ensuring lightweight design, demonstrating strong generalization ability and practical potential for engineering applications.

### Structure-aware cross-directional pooling

**Fig 3:**
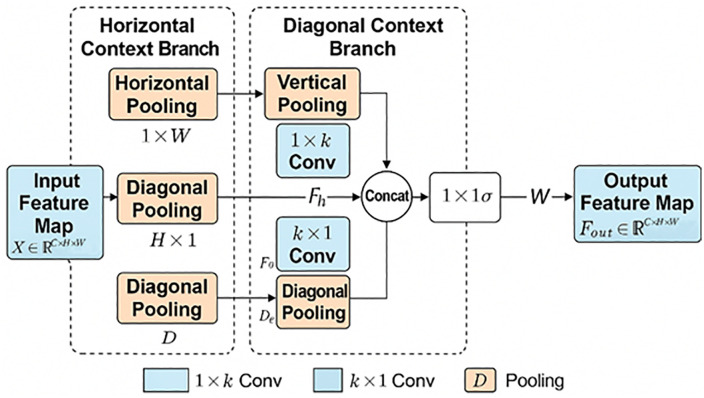
Structure diagram of the SACP module.

To further enhance the structural modeling capability of the network in the segmentation of cracks in ancient wooden structures, we propose a Structure-Aware Cross-directional Pooling (SACP) module. Building upon conventional horizontal and vertical strip pooling, the SACP introduces an additional diagonal strip pooling strategy, thereby enabling joint modeling along horizontal, vertical, and diagonal directions. This design allows the network to better capture elongated cracks, oblique textures, and local structural details, while maintaining low computational overhead. The structure of the SACP module is illustrated in Fig 3.

In conventional spatial modeling, convolution operations are restricted by fixed receptive fields, making it difficult to represent long-range structural dependencies. This limitation becomes more pronounced when cracks exhibit irregular oblique or tortuous patterns, leading to insufficient boundary and structural perception. Strip Pooling [34] partially alleviates this issue by applying horizontal (H × 1) and vertical (1 × W) pooling, which enhances directional modeling to some extent. However, its effectiveness remains limited in scenarios with complex and diverse crack patterns.

To this end, the SACP module introduces a diagonal strip pooling mechanism, which allows the module to simultaneously extract long-range contextual information in the horizontal, vertical, and diagonal directions. This allows the module to perceive crack boundaries and structural extension trends over a larger spatial range, enhancing spatial dependency modeling capabilities. Given an input feature map X ∈ R^C×H×W^, three pooling operations are applied to it:

Horizontal Strip Pooling:


$$\begin{array}{c} y_{i}^{h}=\frac{\text{1}}{W}\sum {\mathrm{\backslash nolimits}}_{j=\text{1}}^{W}x_{i,j} \end{array}$$ (1)

Vertical Strip Pooling:


$$\begin{array}{c} y_{j}^{\text{v}}=\frac{\text{1}}{H}\sum {\mathrm{\backslash nolimits}}_{i=\text{1}}^{H}x_{i,j} \end{array}$$ (2)

Diagonal Strip Pooling:

Let D_k_ denote the set of elements on the k-th diagonal, then


$$\begin{array}{c} y_{k}^{\text{d}}=\frac{\text{1}}{\left|D_{k}\right|}\sum {\mathrm{\backslash nolimits}}_{(i,j)\in D_{k}}^{H}x_{i,j} \end{array}$$ (3)

where D_k_ includes indices from either the top-left to bottom-right or the top-right to bottom-left direction.

The SACP module consists of five key steps:

① **Pooling in three directions**: apply horizontal, vertical, and diagonal pooling to generate Y_h_ ∈R^C×H×1^, Y_v_ ∈R^C×1×W^, and Y_d_ ∈R^C×D^;
② **1D convolutional enhancement**: feed each pooled feature into a 1D convolution layer (kernel size = 3) for context modeling and feature refinement;
③ **Feature expansion and alignment**: restore pooled features to the original spatial dimensions (H × W) via broadcasting, obtaining three directional response maps;
④ **Fusion and activation**: concatenate the response maps along the channel dimension, fuse them with a 1 × 1 convolution, and apply a Sigmoid activation to generate a weight map W ∈ R^C×H×W^;
⑤ **Weighted output**: the final output is computed as:

$$\text{Z}=\text{X}\cdot \sigma (\text{f}({\text{Y}}_{\text{h}}⊕{\text{Y}}_{\text{v}}⊕{\text{Y}}_{\text{d}}))$$ (4)

where ⊕ denotes concatenation, f(⋅) represents the fusion convolution, σ is the Sigmoid function, and ⋅ indicates element-wise multiplication.

The characteristics of the SACP module are summarized in Table 1.

**Table 1:** Characteristics of the SACP module.

Feature	Description
Multi-directional perception	Integrates horizontal, vertical, and diagonal context information to adapt to cracks with diverse shapes
Efficient and lightweight	Uses pooling + 1D convolution + fusion, avoiding complex attention mechanisms and enabling easy deployment
Strong structural modeling	Captures long-range dependencies in multiple directions, improving segmentation of slender and intersecting cracks
Originality	Unlike mainstream modules, SACP introduces diagonal strip pooling, offering an innovative structural modeling approach

In summary, the SACP module simulates the realistic propagation of cracks, which often extend along horizontal–vertical–diagonal paths. By enhancing structural perception while maintaining low computational cost, SACP significantly improves the segmentation performance for slender, discontinuous, and intersecting cracks, thus providing a robust foundation for the analysis of ancient timber structures.

### Multi-route feature extraction

**Fig 4:**
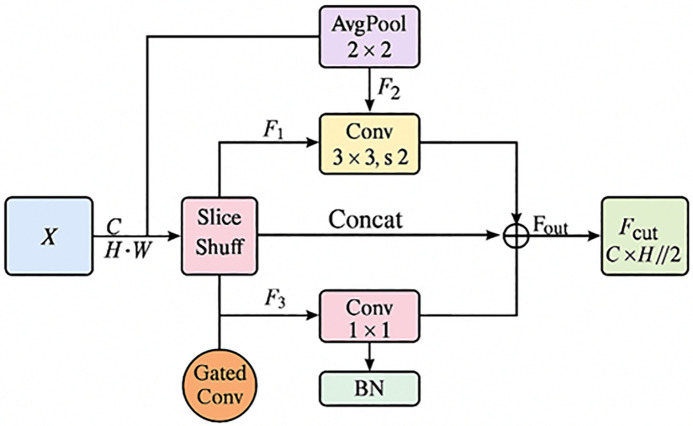
Structure diagram of the MRFE module.

In image segmentation tasks, conventional downsampling operations effectively reduce computational costs but often lead to the loss of fine details, particularly small-scale features such as slender cracks. This loss compromises segmentation accuracy. To address this limitation, we propose a Multi-Route Feature Extraction (MRFE) module (Fig 4), designed as a novel shallow feature downsampling strategy. MRFE enhances robustness and preserves crack details by jointly extracting and compressing features through multiple complementary pathways.

The MRFE module integrates four structurally diverse routes, each focusing on different aspects of feature modeling, including spatial distribution, multi-scale context, and content guidance. Given an input feature map X ∈R^C×H×W^, the process is as follows:

① **Main convolutional path**: standard downsampling using a 3 × 3 convolution with stride 2:

$${\text{F}}_{\text{1}}={\text{Conv}}_{\text{3}\times \text{3},\text{s}=\text{2}}(\text{X})$$ (5)

② **Smoothing-pooling path**: 2 × 2 average pooling followed by a 1 × 1 convolution to enhance structural information:

$${\text{F}}_{\text{2}}={\text{Conv}}_{\text{1}\times \text{1}}\;\left({\text{AvgPool}}_{\text{2}\times \text{2}}(\text{X})\right)$$ (6)

③ **Slicing–channel shuffle path**: drawing on the idea of channel shuffling, the input feature map is first sliced into four non-overlapping sub-regions C_1_, C_2_, C_3_, and C_4_ in a 2 × 2 fashion, each of size H/2 × W/2. These regions are then concatenated along the channel dimension and shuffled to achieve a more even distribution of features and enhance the network’s sensitivity to local details. The process flow is as follows:

$${\text{F}}_{\text{4}}=\text{GatedConv}(\text{X})$$ (7)

After the four slice regions are concatenated in the channel dimension, a 1 × 1 convolution operation is performed to reduce the dimension to reduce the number of parameters and form the feature output F_3_.

④ **Gated convolutional path**: a learnable gated convolution emphasizes critical regions such as crack boundaries and distinctive textures while suppressing redundant information:

$${\text{F}}_{\text{4}}=\text{GatedConv}(\text{X})$$ (8)

The outputs from the four parallel paths are concatenated along the channel dimension, followed by a 1 × 1 convolution for dimensionality reduction and batch normalization (BN) for feature standardization. This process ultimately produces a robust downsampled feature representation enriched with contextual information.


$${\text{F}}_{\text{out}}=\text{BN}\left({\text{Conv}}_{\text{1}\times \text{1}}\;\left(\text{Concat}\left({\text{F}}_{\text{1}}{,\text{F}}_{\text{2}},{\text{F}}_{\text{3}},{\text{F}}_{\text{4}}\right)\right)\right)$$ (9)

The fusion process can be expressed as:


fusion←BN(Conv(Concat(x,y,z…))) (10)

The characteristics of the MRFE module are summarized in Table 2.

**Table 2:** Characteristics of the MRFE module.

Feature	Description
Stronger robustness	Multi-route enhancement to suppress noise
Crack-preserving ability	Retains weak textures and fine cracks
Better adaptability	Different routes capture features of varying scales and types
Low computational cost	Depthwise convolution and 1 × 1 compression

In summary, the MRFE module leverages a multi-route parallel extraction strategy to simulate the diverse patterns of cracks and edge details across scales and orientations. By integrating four complementary paths—standard convolution, smoothing pooling, slicing–reconstruction, and gated convolution—MRFE enhances the robustness and diversity of shallow features while maintaining computational efficiency. This provides a stable and reliable foundation for higher-level semantic modeling and significantly improves the segmentation accuracy and reliability of cracks in ancient timber structures.

### Guided semantic-spatial fusion module

**Fig 5:**
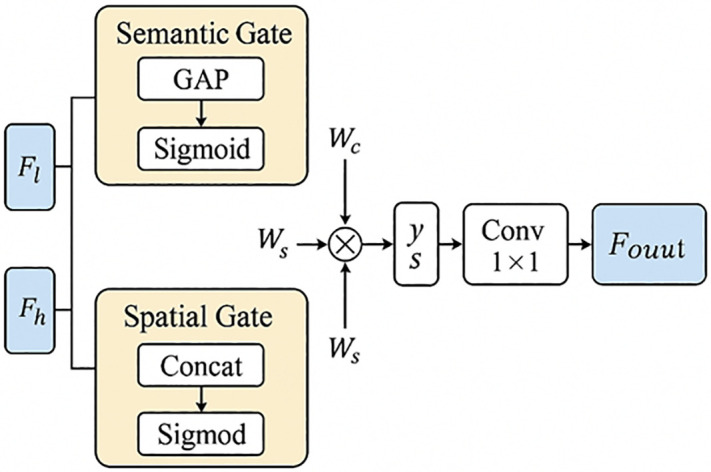
Structure diagram of the GSSFusion module.

To enhance the ability of the model to preserve fine details and capture contextual information in crack images of ancient wooden structures, we propose the Guided Semantic-Spatial Fusion Module (GSSFusion), as illustrated in Fig 5. This module integrates semantic and spatial guidance mechanisms to selectively fuse features from different scales and semantic levels, thereby achieving more accurate crack boundary perception and detail restoration.

The module takes as input the low-level feature map F_l_ ∈ R^Cl×H×W^ from the encoder and the high-level feature map F_h_ ∈ R^Ch×H×W^ from the decoder. The fusion process of GSSFusion consists of three main steps:

① **Semantic Channel Guidance:** Global average pooling (GAP) is applied to extract global semantic information from F_h_. Two cascaded 1 × 1 fully connected layers (equivalent to convolutions) are then used to generate channel attention weights W_C_ ∈ R^Cl×1 × 1^, which emphasize informative channels in F_l_:

$$W_{\text{c}}=\;\sigma ({\text{Conv}}_{\text{1}\times \text{1}}^{\left(\text{2}\right)}(\text{ReLU}({\text{Conv}}_{\text{1}\times \text{1}}^{\left(\text{1}\right)}(\text{GAP}(F_{\text{h}})))))$$ (11)

The channel weights are subsequently applied to rescale F_l_:


$${\text{F}}_{\text{l}}^{\prime }=F_{\text{l}}*W_{\text{c}}$$ (12)

② **Spatial Position Guidance:** To further integrate spatial positional information, F_l_ and F_h_ are concatenated along the channel dimension and processed with a 7 × 7 convolution to generate a spatial attention map Ws ∈ R^1×H×W^:

$${\text{W}}_{\text{S}}=\sigma \left({\text{Conv}}_{\text{7}\times \text{7}}\left(\text{Concat}\left({\text{F}}_{\text{1}},{\text{F}}_{\text{h}}\right)\right)\right)$$ (13)

This spatial attention map is then applied to the channel-guided feature:


$${\text{F}}_{\text{1}}^{\prime \prime }={\text{F}}_{\text{l}}^{\prime }*W_{\text{s}}$$ (14)

③ **Residual Fusion and Compressed Output: Finally**, F_l_′′, the original low-level feature F_l_, and the high-level feature F_h_ are concatenated along the channel dimension. A 1 × 1 convolution followed by batch normalization (BN) is then applied to compress and normalize the fused representation:

$${\text{F}}_{\text{out}}=\text{BN}\left({\text{Conv}}_{\text{1}\times \text{1}}\left(\text{Concat}\left({\text{F}}_{\text{1}}^{\prime \prime },{\text{F}}_{\text{1}}{,\text{F}}_{\text{h}}\right)\right)\right)$$ (15)

Among them, σ represents the Sigmoid activation function, Conv represents the convolution operation, Concat represents the concatenation in the channel dimension, and BN represents the batch normalization operation.

The characteristics of the GSSFusion module are summarized in Table 3.

**Table 3:** Characteristics of the GSSFusion module.

Feature	Description
Lightweight design	Utilizes a GAP + MLP structure without complex Transformer or multi-head mechanisms
Dual-guided fusion	Combines high-level global semantic attention with spatial position response to enhance crack regions
Residual robustness	Incorporates a residual path with low-level features to mitigate shallow information loss
Multi-scale adaptability	Improves adaptability to complex backgrounds and fine crack microstructures in ancient buildings

By leveraging a lightweight GAP-MLP structure for semantic guidance, integrating spatial position responses, and introducing a residual path to mitigate shallow feature loss, GSSFusion effectively enhances crack boundary representation and detail recovery. Overall, this module achieves cross-level and multi-perspective guided feature fusion, significantly improving adaptability to complex backgrounds and fine crack patterns in ancient building crack detection tasks.

## Dataset

### Image acquisition

To address the challenge of limited data, a crack dataset of ancient wooden structures was constructed, covering complex backgrounds and diverse crack types. Images were collected from representative Minnan-style historic buildings in Nanjing County (Zhangzhou City) and Liancheng County (Longyan City), Fujian Province, with all photographs taken on-site by the authors. To ensure consistency, the same camera model with fixed focal length, distance, and angle was used. The original resolution was 3280 × 2464 pixels, and 256 high-quality images were carefully selected as the initial dataset.

**Fig 6:**
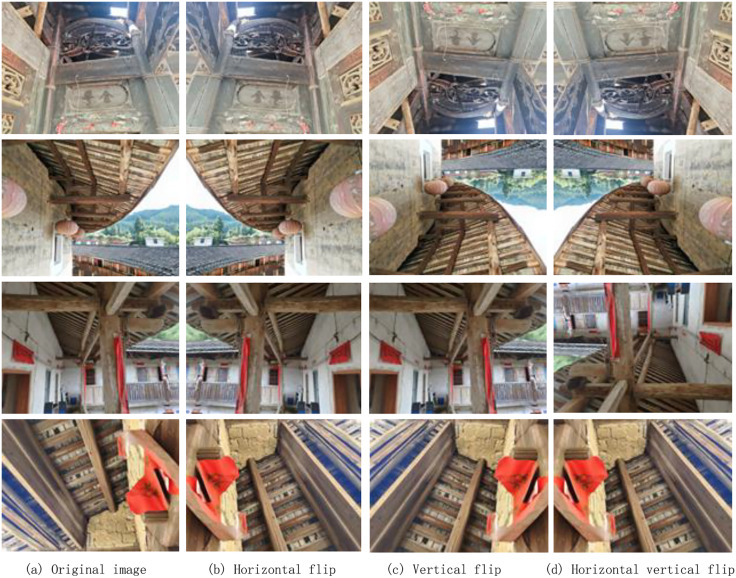
Schematic diagram of image data enhancement.

To enlarge the dataset, augmentation techniques such as horizontal, vertical, and combined flipping rotation were applied (see Fig 6). A total of 144 new images were generated, expanding the dataset to 400 RGB images labeled systematically as crackspread1–crackspread400. During dataset partitioning, each original image and its augmented versions were placed in the same subset to avoid data leakage, thereby ensuring independence across training, validation, and test sets. This strategy improved the model evaluation and guaranteed the reproducibility of the experiments.

### Image cropping

**Fig 7:**
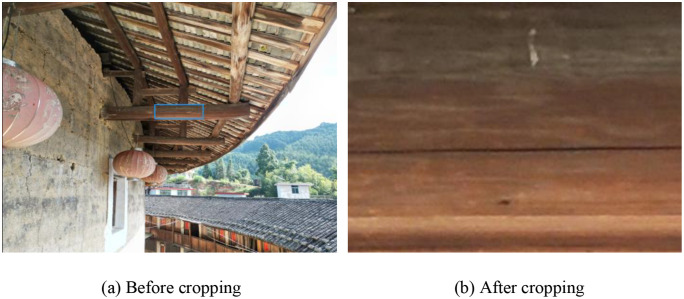
Schematic diagram of image data enhancement. (a) Before cropping (b) After cropping.

To reduce computational overhead and improve the recognition of fine-scale cracks, an image cropping strategy was applied. Crack-prone regions were first manually identified, and automated cropping was then performed using Python to extract the relevant areas while applying boundary checks to prevent information loss at the edges. The cropped images were uniformly resized to 640 × 480 pixels and systematically named crackcut1–crackcut400. As shown in Figs 7(a) and 7(b), this process effectively transformed large raw images into standardized crack-focused inputs, reducing redundancy and enhancing feature salience for subsequent model training.

### Image grayscale

After cropping the image, the resulting image remains in BMP format, which is an RGB true-color image. An RGB image consists of three channels: red (R), green (G), and blue (B), with each channel’s grayscale value ranging from 0 to 255. Since RGB images contain three channels, they require more memory resources for processing, which results in relatively slower processing speeds. In contrast, grayscale images contain only one channel, with the number of colors limited to 255. When the values of R, G, and B are all 255, the image appears white; when these values are all 0, the image appears black. Processing grayscale images significantly reduces computational load, thereby improving processing speed. Given that the subsequent algorithm only requires grayscale images, we have converted the crack images to grayscale to enhance detection efficiency. Common grayscale processing methods include the maximum value method, the average method, and the weighted average method, each with its own advantages and disadvantages, as detailed in Table 4. By transforming RGB to grayscale, the data volume is effectively reduced without losing image information, and the subsequent crack segmentation process is optimized.

**Table 4:** Comparison of grayscale conversion algorithms.

Method	Advantage	Disadvantages
**Maximum Method**	The brightest part of the image is used to emphasize brightness contrast. The maximum value of the three RGB color channels is taken as the grayscale value Gray = max(R,G,B)	Some details may be lost because only the maximum value in RGB is considered, not the information of all color channels.
**Average method**	Averaging the values of the three RGB color channels can provide a more balanced grayscale value, which is suitable for situations where the color distribution in the image is relatively uniform.	May miss some details because it simply averages the RGB values and does not take into account the human eye’s sensitivity to different colors
**Weighted average method**	Considering that the human eye has different sensitivities to different colors, a weight of 0.299R + 0.587G + 0.114*B is used to retain more details.	The calculation is relatively complex and may be slower than the maximum and average methods.

After analyzing the three grayscale conversion methods, the weighted average method was deemed the optimal choice for segmenting cracks in ancient timber structures. This method first obtains the image’s height and width, then creates a new grayscale image and loads the pixel data to compute the weighted average. The weighted average method was prioritized because it more accurately simulates the human eye’s color perception, which is crucial for maintaining the contrast between the cracks and surrounding wood, thereby making the cracks easier to identify. Furthermore, the weighted average method better preserves details when processing images with complex textures and color variations, which is particularly important for crack detection. In practical applications, the weighted average method has proven to be highly effective due to its widespread use in image processing and computer vision, especially in scenarios where accuracy and detail preservation are critical. Therefore, the weighted average method was selected for the grayscale conversion in this study to ensure the accuracy and efficiency of crack segmentation.

### Image noise reduction

Electronic devices, such as cameras, often introduce noise into images due to the influence of circuit structures and transmission mediums. Common types of noise include salt-and-pepper noise, characterized by randomly scattered black and white pixels, and Gaussian noise, which appears as blurred spots. Since it is impossible to completely eliminate noise, various denoising methods were assessed to identify the most effective approach for the algorithm in this study. The denoising techniques evaluated for removing cracks in ancient timber structures include median filtering, mean filtering, Gaussian filtering, bilateral filtering, non-local means filtering (NL-means), and block-matching 3D filtering (BM3D). A detailed analysis of these methods and their results is provided in Table 5.

**Table 5:** Comparison of noise reduction algorithms.

Method	Advantage	Disadvantages
**Median filter**	Effective for salt-and-pepper noise; preserves edges	Less effective for Gaussian noise
**Mean filter**	Simple, low-cost implementation	Blurs edges and fine details
**Gaussian filter**	Smooths Gaussian noise with distance-based weights	May oversmooth edges; slower computation
**Bilateral filter**	Removes noise while preserving edges	High computational cost; less effective on large regions
**NL-means**	Exploits patch similarity for detail-preserving denoising	Very slow; high computational cost
**BM3D**	Superior noise reduction while retaining fine structural details	Complex, time-consuming implementation

The BM3D algorithm, known for its excellent denoising performance and effective preservation of image details, is widely considered the preferred method for crack detection in ancient timber structures. This algorithm processes 2D images using block matching and 3D transformation techniques, without relying on the 3D coordinates of the image. The workflow involves dividing the image into small blocks, finding similar patches, and applying 3D transformation for denoising. Specifically, the BM3D algorithm aggregates similar image blocks in the 3D transform domain to more accurately estimate the true signal, effectively removing noise while preserving image details. Although BM3D has a high computational complexity, ensuring data quality is crucial when constructing datasets. To reduce irrelevant information and minimize the algorithm’s computational load, the images are first converted to grayscale. This preprocessing step enhances the efficiency of subsequent annotation and training processes. For instance, the BM3D algorithm is applied for denoising by calling the function apply_denoising_methods(image_path, output_folder) and using bm3d = cv2.fastNlMeansDenoisingColored(image, None, 10, 10, 7, 21) for processing. Finally, the images are renamed using os.rename(old_file_path, new_file_path) to facilitate further processing. Therefore, this study adopts the BM3D algorithm for denoising, reducing the computational burden during model training.

### Image annotation

To ensure the safety and stability of timber structures, the classic image annotation tool Labelme was used to accurately delineate the crack contours. The annotation information was saved in JSON format, which includes the image name, line and fill color, target names, and the location data of the annotation points. After completing the data annotation, Python was used to convert the JSON files into grayscale mask images. This process involved iterating through all JSON files, extracting image width and height to create new images with a black background. The shapes labeled as “wood” were then selected, and their vertex coordinates were extracted and converted to integers before being drawn onto the new image. These mask images (or ground truth images) provide pixel-level annotation information for each semantic class in the original image, which is crucial for model training. The 400 annotated images were stored in the “masks” folder, while the corresponding 400 preprocessed training images were stored in the “images” folder.

**Fig 8:**
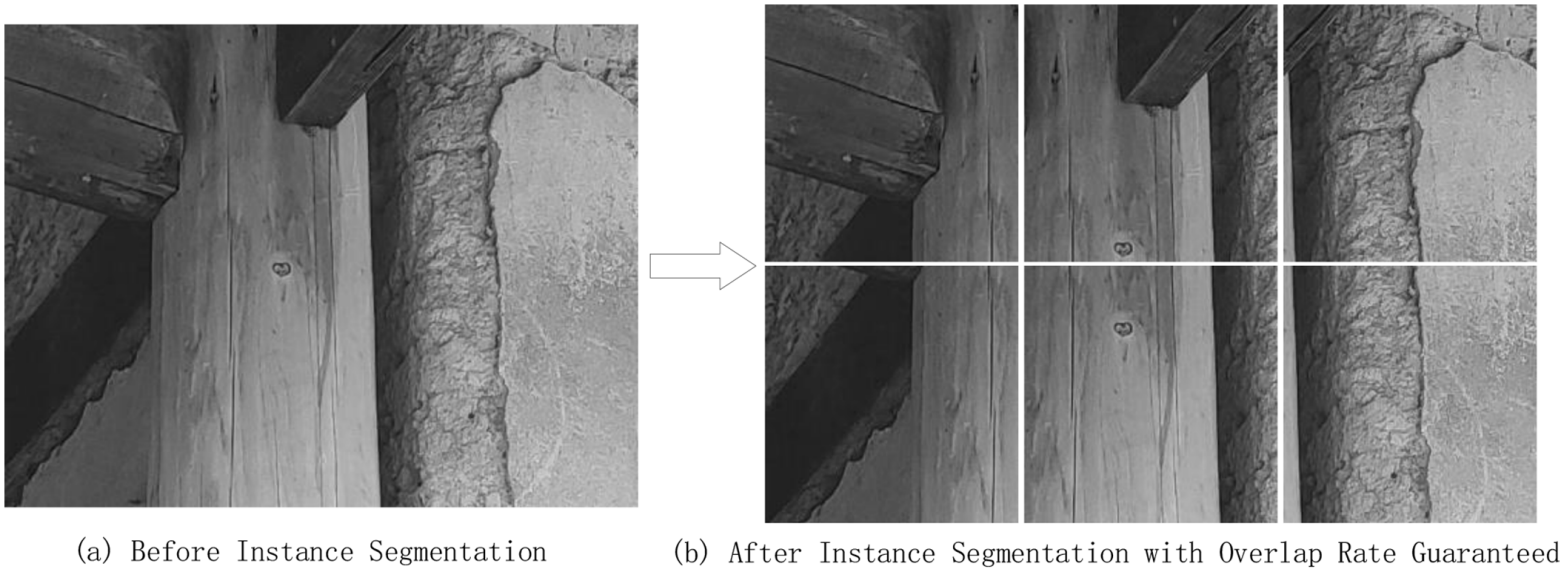
Instance segmentation effect diagram after ensuring the overlap rate. (a) Before Instance Segmentation (b) After Instance Segmentation with Overlap Rate Guaranteed.

Considering the high resolution and large amount of irrelevant information in the original images, direct training yielded suboptimal results. Additionally, the crack detection dataset suffers from class imbalance, with the background class dominating the pixel count and the crack pixels being relatively few. This causes the network to become overly confident in predicting the background class, leading to misclassification of cracks and a large number of false negatives. To address this, an instance segmentation method was employed, as shown in Fig 8. The 640 × 480 crack images were split into six 256 × 256 sub-images with a certain overlap, ensuring continuity and completeness in the image segmentation process.

This segmentation process was implemented using Python programming. The specific steps are as follows: first, the effective overlapping region size was calculated to determine the required number of image patches; then, the images were iteratively cropped, and the left, top, right, and bottom coordinates of each patch were computed to ensure that the cropping operation remained within the image boundaries while appropriately handling edge cases. Finally, all image files were traversed, cropped, and saved for subsequent processing.

Through this method, a total of 2,400 sub-images were generated. To avoid data leakage, strict constraints were imposed during dataset partitioning: all sub-images derived from the same original image and its augmented versions were assigned to the same subset, ensuring that they did not simultaneously appear in the training, validation, and test sets. Ultimately, the dataset was divided into training, validation, and test sets at a ratio of 7:2:1, containing 1,680, 480, and 240 images, respectively. This partitioning strategy not only improved the reliability of model training and evaluation but also mitigated evaluation bias caused by overlapping data. Detailed information on the experimental dataset after partitioning is provided in Table 6. The dataset, together with the source code, has been uploaded to the GitHub repository, allowing researchers to directly reproduce the data processing and partitioning procedures.

**Table 6:** Details and metrics of networks used for image classification.

Category	Total	Train	Val	Test	Resolution	Category
Crack Image	2400	1680	480	240	256*256	Crack Image

## Model training

### Experimental environment and parameter settings

The dataset construction, model training, and testing were conducted on a single machine. The experimental setup includes both hardware and software components. The hardware consists of a device with an Intel(R) Core(TM) i7-9750H processor and 16GB of RAM to support efficient data processing and model training. For the software, PyTorch 1.13.1, optimized for CUDA 11.7, was used as the deep learning framework to leverage the computational capabilities of NVIDIA GPUs. PyCharm was selected as the integrated development environment, with Python 3.8.19 for code compilation. A detailed configuration of the experimental environment is shown in Table 7. This setup ensures a stable and efficient environment for the experiments.

**Table 7:** Experimental environment.

Category	Parameters
System	Windows 10 Home
CPU	Intel(R) Core(TM) i7-9750H with 16GB Memory
GPU	NVIDIA GeForce RTX 2060
Deep Learning Frameworks	Pytorch(1.13.1 + cu117)
Library	OPENCV, CUDNN, CUDA

During the training of the crack segmentation network, factors such as computational resources and model performance are carefully considered to ensure sufficient training cycles. The model undergoes at least 150 epochs, or until the F1 score on the validation set plateaus. The initial learning rate (lr) is set to 0.001, and the CosineAnnealingLR scheduler is used with a maximum iteration number (T_max) of 50 and a minimum learning rate (eta_min) of 0.00001 [35]. A batch size of 16 samples is employed. To optimize the training process, the AdamW optimizer is selected [36]. As an enhanced version of the Adam algorithm, AdamW introduces weight decay regularization. It directly applies weight decay to the weights, preventing the momentum from affecting the regularization coefficient, which leads to faster convergence compared to the standard Adam optimizer.

### Model evaluation metrics

In the semantic segmentation task of cracks in wooden components of ancient buildings, the goal is to accurately classify each pixel in the image as a crack pixel or a background pixel. By performing pixel-level classification, a confusion matrix can be generated and the segmentation results can be analyzed in detail. Common indicators include:

① True Positive (TP): the number of pixels correctly predicted as cracks;
② True Negative (TN): the number of pixels correctly predicted as background;
③ False Positive (FP): the number of pixels incorrectly predicted as cracks;
④ False Negative (FN): the number of pixels incorrectly predicted as background.

To address the challenge of crack size measurement in the crack segmentation task, various loss functions are compared to evaluate their impact on the network performance. Finally, BceDiceLoss was selected as the optimal solution. BceDiceLoss combines binary cross entropy loss (BCELoss) and Dice loss to calculate loss values at different stages. The design of this composite loss function helps to significantly improve the performance of the network in crack segmentation tasks. The specific loss function formulas are as follows (16) and (17):


$$L_{i}=Bce(\text{y},\hat{y})+Dice(y,\hat{y})$$ (16)

$$\begin{array}{c} \zeta =\sum {\mathrm{\backslash nolimits}}_{i=\text{0}}^{\text{5}}\lambda_{i}*l_{i} \end{array}$$ (17)

To comprehensively assess the model’s performance, several key metrics, including the number of parameters, accuracy, and various evaluation indicators, are calculated. Accuracy represents the proportion of correctly classified pixels relative to the total number of pixels. The F1 score combines precision and recall (or sensitivity) to measure the balance between the model’s accuracy and completeness. In semantic segmentation tasks, the model’s segmentation quality is typically evaluated using Intersection over Union (IoU) and Pixel Accuracy (PA). IoU is a crucial metric in object detection and semantic segmentation, as it measures the accuracy of the model by calculating the ratio of the intersection and union of the predicted and true areas. The Mean Intersection over Union (mIoU) reflects the average overlap between the segmented and target areas; higher mIoU values indicate better segmentation performance. Pixel Accuracy (PA) denotes the proportion of correctly identified crack pixels among all crack pixels. Additionally, specificity assesses the model’s ability to correctly classify non-crack (background) pixels, while sensitivity measures its capacity to accurately identify crack pixels. Together, specificity and sensitivity provide a comprehensive view of the model’s performance in crack segmentation. The formulas for these metrics are as follows:


$$P_{IoU}=\frac{N_{TP}}{N_{TP}+N_{FP}+N_{FN}}$$ (18)

$$P_{PA}=\frac{N_{TP}+N_{TN}}{N_{TP}+N_{FP}{+N}_{TN}+N_{FN}}$$ (19)

$$P_{Sp}=\frac{N_{TN}}{N_{TN}+N_{FP}}$$ (20)

$$P_{Se}=\frac{N_{TP}}{N_{TP}+N_{FN}}$$ (21)

In this context, N_TP_ refers to the number of true positive samples, representing the samples correctly classified as positive by the model. N_FP_ denotes the number of false positive samples, which are negative samples incorrectly predicted as positive. N_FN_ represents the number of false negative samples, which are positive samples that the model failed to identify. Lastly, N_TN_ refers to the number of true negative samples, which are correctly classified as negative by the model.

### Experimental result

To comprehensively verify the effectiveness and generalization ability of the proposed model, systematic comparative experiments were conducted on both the self-constructed wooden component crack dataset and the publicly available Masonry dataset [37]. All experiments were carried out under identical training parameters, data augmentation strategies, and hardware configurations to strictly control variables and ensure fairness and reproducibility. The benchmark models for comparison included representative U-Net variants (AttU-Net [18], ABCNet [19], CMUNet [20]), lightweight networks (ENet [21], A2FPN [22]), as well as advanced architectures that have recently demonstrated strong performance in visual segmentation tasks, such as SegFormer [31], Swin-Unet [32], and the crack-optimized SMG-Net.

To reduce the influence of randomness, each model was independently trained and tested 10 times, and the average results after excluding outliers were reported as the final performance indicators. Furthermore, to validate the robustness of the results, statistical analyses including ANOVA and t-tests were conducted across the primary evaluation metrics (loss, mIoU, F1-score, accuracy, specificity, and sensitivity). The results demonstrated that the differences between SMG-Net and most baseline models were statistically significant at the 95% confidence level (p < 0.05).

**Fig 9:**
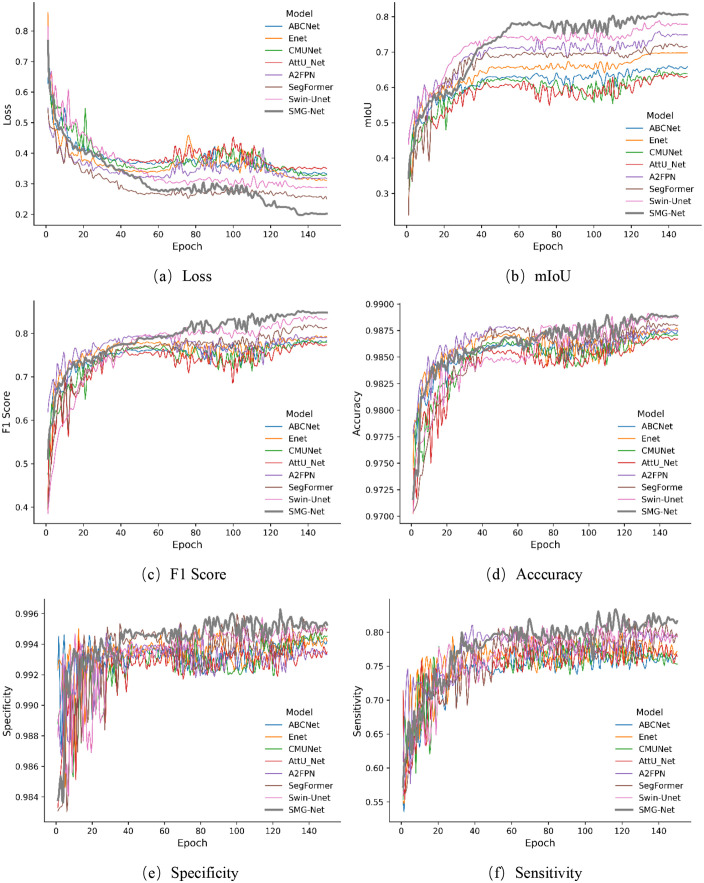
Comparison of training processes for six evaluation metrics on the wood structure crack dataset. (a) Loss, (b) mIoU, (c) F1 Score (d) Acccuracy, (e) Specificity, (f) Sensitivity.

**Fig 10:**
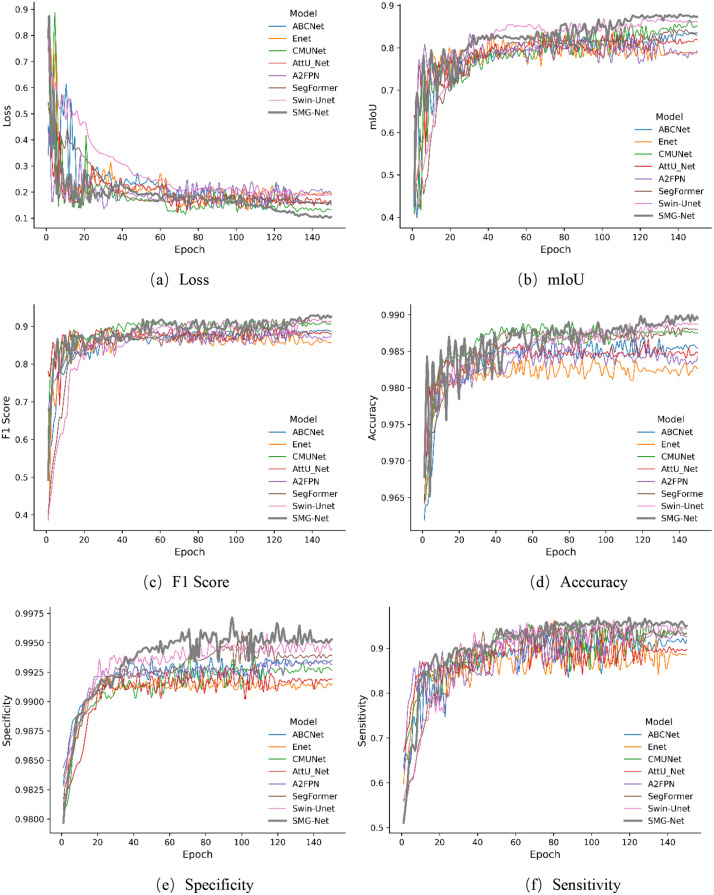
Comparison of training processes for six evaluation metrics on the masonry dataset. (a) Loss, (b) mIoU, (c) F1 Score, (d) Acccuracy, (e) Specificity, (f) Sensitivity.

**Fig 11:**
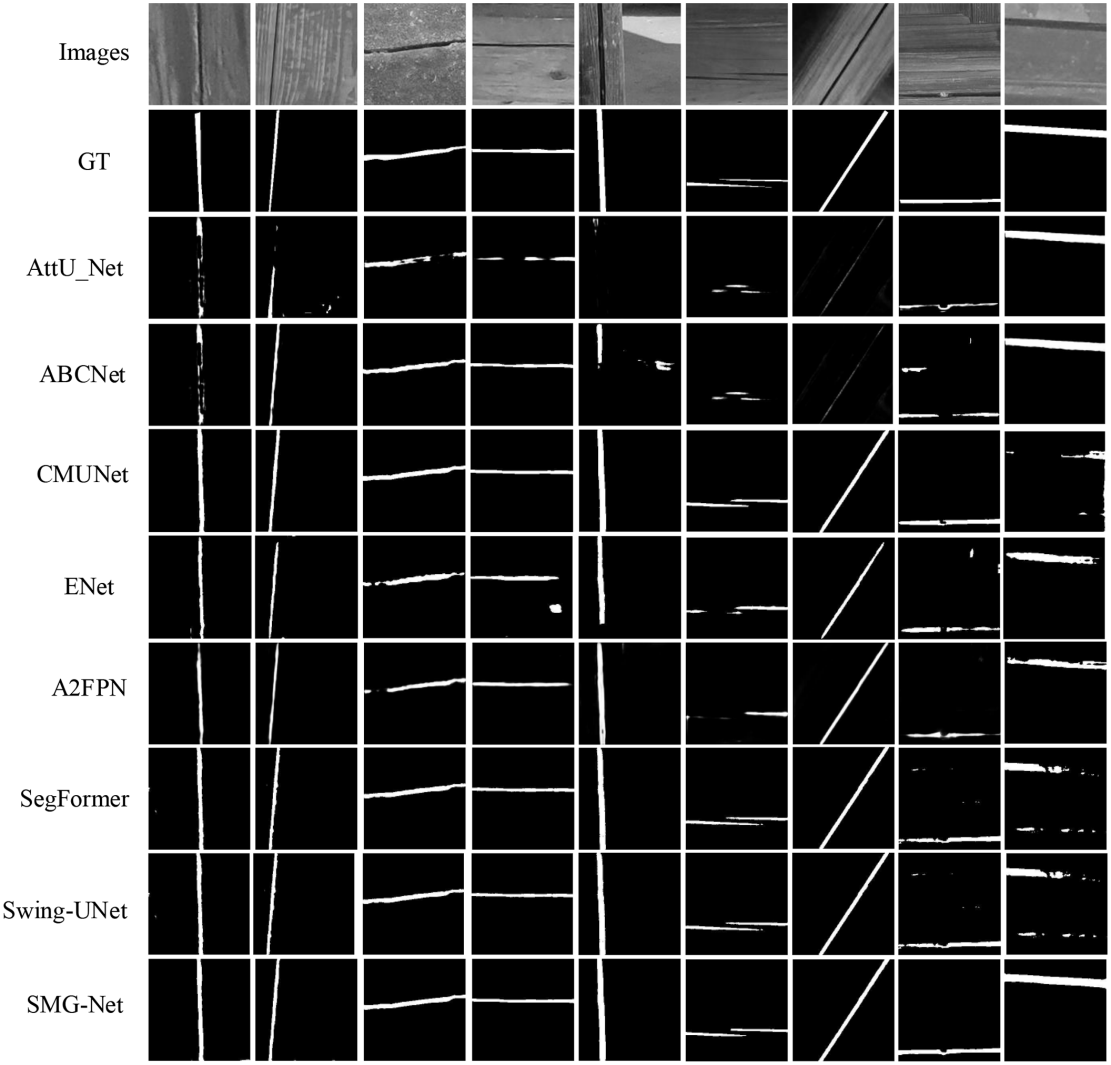
Comparison of results for six network architectures on the wood structure crack dataset.

**Fig 12:**
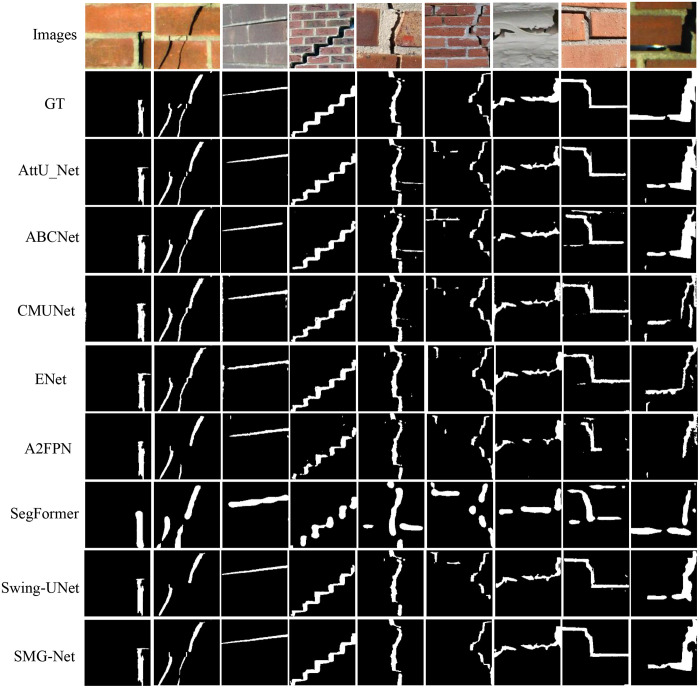
Comparison of results for six network architectures on the masonry dataset.

Figs 9 and 10 illustrate the performance trajectories of different models on the wooden crack dataset and the Masonry dataset, respectively, across the six evaluation metrics during training. It can be observed that SMG-Net exhibits superior convergence speed and stability. Figs 11 and 12 present typical qualitative segmentation results on both datasets, which further highlight the advantages of SMG-Net in accurately delineating crack boundaries and preserving fine details.

Tables 8 and 9 summarize the average results of ten independent runs. On the wooden crack dataset, SMG-Net achieved the best performance in terms of loss, mIoU, F1-score, and sensitivity, with an mIoU of 0.8112, an F1-score of 0.8513, and a sensitivity of 0.8310, outperforming advanced models such as SegFormer and Swin-Unet. On the Masonry dataset, SMG-Net likewise demonstrated strong superiority, achieving an mIoU of 0.8791, an F1-score of 0.9301, and a sensitivity as high as 0.9692, all significantly better than the other comparative models. Notably, SMG-Net maintained stable segmentation accuracy even on samples with small cracks or heavy noise interference, indicating strong robustness and generalization capacity.

**Table 8:** Comparative experimental results on the wood structure crack dataset.

Models	loss	mIoU (%)	F1/%	accuracy/%	specificity/%	sensitivity/%
**AttU_Net**	0.3461	0.6382	0.7791	0.9869	0.9953	0.7981
**ABCNet**	0.3312	0.6613	0.7851	0.9875	0.9946	0.7887
**CMUNet**	0.3242	0.6499	0.7879	0.9872	0.9953	0.7947
**ENet**	0.3112	0.6980	0.7938	0.9877	0.9956	0.8034
**A2FPN**	0.3146	0.7589	0.7958	0.9879	0.9952	0.8132
**SegFormer**	0.2502	0.7231	0.8191	0.9884	0.9960	0.8215
**Swin-Unet**	0.2846	0.7889	0.8391	0.9890	0.9958	0.8193
**SMG-Net**	0.1974	0.8112	0.8513	0.9891	0.9962	0.8310

**Table 9:** Comparative experimental results on the masonry dataset.

Models	loss	mIoU (%)	F1/%	accuracy/%	specificity/%	sensitivity/%
**AttU_Net**	0.1209	0.8380	0.8961	0.9867	0.9942	0.9523
**ABCNet**	0.1343	0.8379	0.8994	0.9869	0.9959	0.9539
**CMUNet**	0.1153	0.8656	0.9196	0.9888	0.9953	0.9574
**ENet**	0.1324	0.8412	0.8899	0.9839	0.9950	0.9592
**A2FPN**	0.1360	0.8293	0.8898	0.9866	0.9949	0.9594
**SegFormer**	0.1502	0.8431	0.9191	0.9884	0.9960	0.9591
**Swin-Unet**	0.1846	0.8703	0.9208	0.9890	0.9958	0.9597
**SMG-Net**	0.1023	0.8791	0.9301	0.9899	0.9969	0.9692

In addition, SMG-Net achieves high segmentation accuracy while maintaining lightweight efficiency. Through the effective combination of depthwise separable convolutions and pointwise convolutions, the model significantly reduces computational complexity and parameter size. Combined with scalable width and resolution multipliers, SMG-Net can adapt to resource-constrained environments without sacrificing performance. These characteristics underscore its strong potential for practical applications in crack detection of ancient wooden structures.

### Ablation experiment

**Fig 13:**
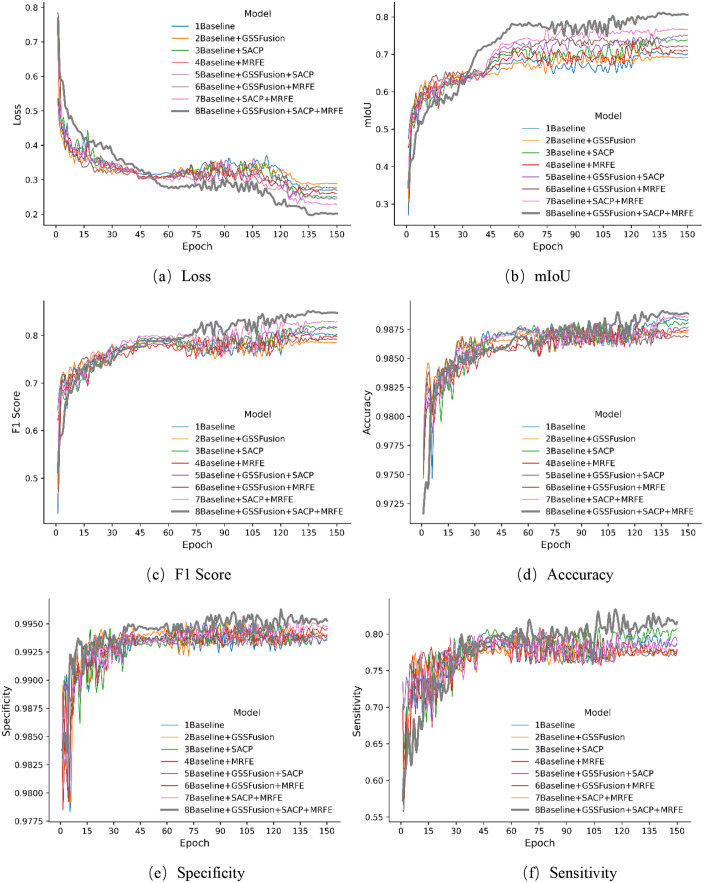
Comparison of the training process of six evaluation indicators in ablation experiments. (a) Loss, (b) mIoU, (c) F1 Score, (d) Acccuracy, (e) Specificity (f) Sensitivity.

**Fig 14:**
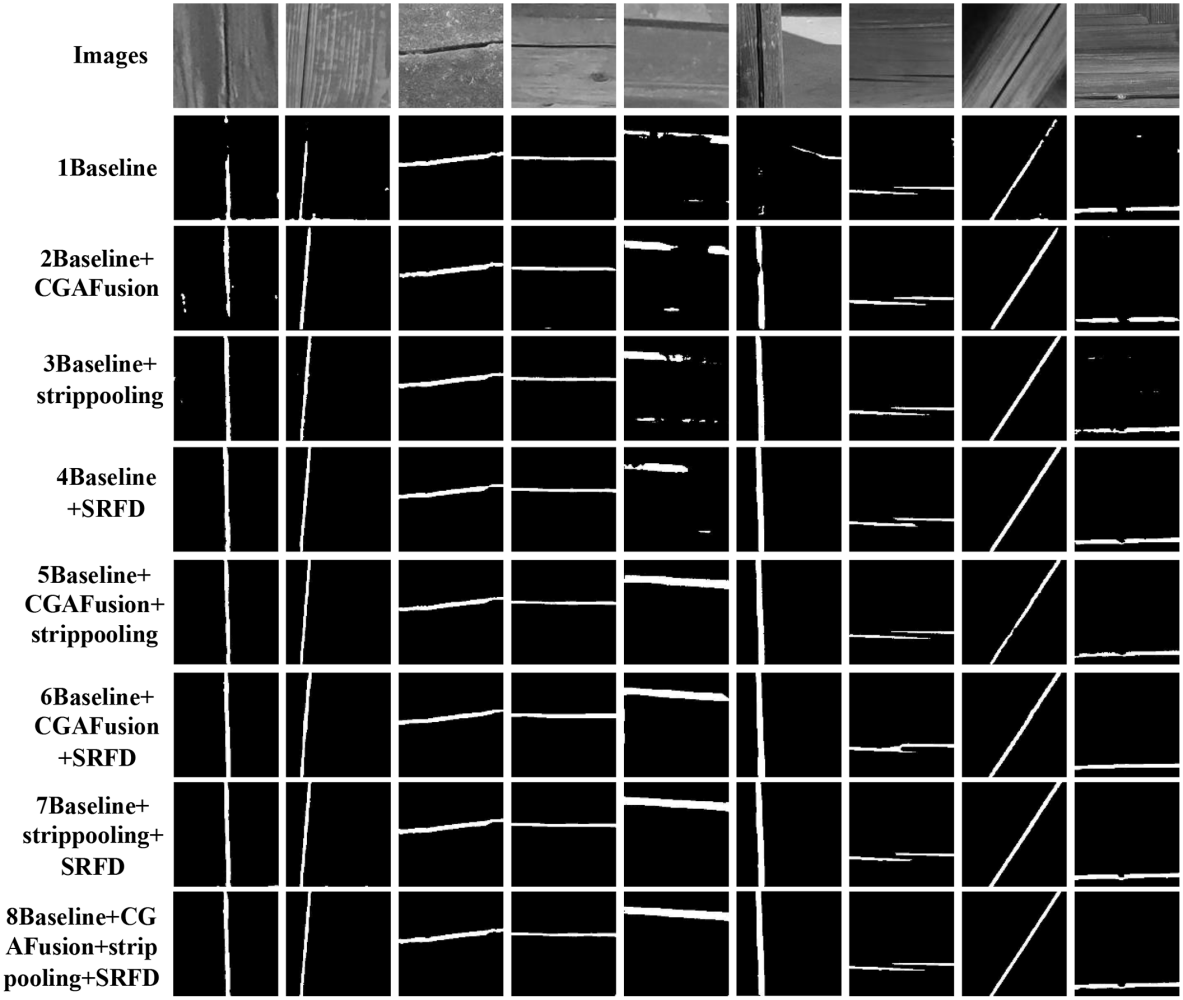
Ablation experiment results after adding different modules.

To clarify the contributions of individual components within SMG-Net, an ablation study was conducted on the wooden crack dataset. Eight experimental configurations were designed by progressively integrating three modules—SACP, MRFE, and GSSFusion—into the baseline model. Each configuration was independently trained 10 times, and mean values were reported to ensure reliability. The results are summarized in Table 10, while Figs 13 and 14 illustrate metric variations during training and representative prediction outcomes.

**Table 10:** Ablation experiment results on the wood component crack dataset.

Modules	loss	mIoU (%)	F1/%	acc/%	spe/%	sen/%
**Baseline**	0.2694	0.6889	0.8077	0.9886	0.9950	0.7989
**Baseline+GSSFusion**	0.2862	0.6684	0.8014	0.9879	0.9954	0.8054
**Baseline+SACP**	0.2477	0.7027	0.8201	0.9882	0.9948	0.8092
**Baseline+MRFE**	0.2504	0.7004	0.8124	0.9880	0.9948	0.7980
**Baseline +GSSFusion+SACP**	0.2432	0.7191	0.8201	0.9880	0.9949	0.8012
**Baseline +GSSFusion+MRFE**	0.2690	0.7067	0.8026	0.9879	0.9950	0.8031
**Baseline+SACP+MRFE**	0.2279	0.7455	0.8300	0.9887	0.9952	0.8034
**Baseline+GSSFusion+SACP+MRFE**	0.1974	0.8112	0.8513	0.9891	0.9962	0.8310

The results demonstrate that the SACP module consistently reduced loss and improved both mIoU and F1-score, indicating its strong ability to capture crack structures. The MRFE module also produced notable gains in mIoU and loss reduction, highlighting its effectiveness in multi-scale feature preservation. In contrast, the GSSFusion module, when introduced alone, did not yield significant benefits and occasionally led to declines. However, when all three modules were integrated, SMG-Net achieved the best overall performance (loss = 0.1974, mIoU = 81.12%, F1 = 85.13%, sensitivity = 83.10%), surpassing all other settings.

From a mechanistic perspective, SACP enhances spatial awareness by incorporating horizontal, vertical, and diagonal strip pooling to adapt to irregular crack patterns. MRFE improves robustness through multi-path downsampling, channel shuffling, and gated convolution, thereby preserving spatial structures while suppressing noise. GSSFusion optimizes cross-layer feature interaction via joint semantic–spatial attention, which, although limited alone, contributes to synergistic improvements when combined with SACP and MRFE.

Overall, the ablation study confirms that SACP and MRFE independently provide substantial improvements, while the joint integration of all three modules maximizes segmentation accuracy and robustness. These findings highlight the importance of collaborative module design in improving the practical applicability of SMG-Net for crack detection tasks.

## Conclusion

This study explored the application of deep learning in crack segmentation of ancient wooden components and proposed an improved lightweight model, SMG-Net. By integrating structural-aware pooling (SACP), multi-resolution feature enhancement (MRFE), and guided spatial–spectral fusion (GSSFusion), the model achieved superior segmentation accuracy and robustness while maintaining efficiency. Experimental results on both the self-built wooden crack dataset and the Masonry dataset demonstrated its strong generalization ability and practical value. Furthermore, the pixel-level segmentation results can be mapped to real-world dimensions for risk assessment based on national standards, providing technical support for the safety monitoring of ancient wooden structures. Future work will focus on further optimizing model design and extending its application to broader scenarios such as digital heritage conservation, inspection, and post-disaster assessment.

## References

[1] Han X, Zhou HB, Huang L, Wang SY, Wang WB. Crack types and characteristics of timber members in ancient building in North China. Chin J Wood Sci Technol. 2024;38(2):1–11. doi: 10.12326/j.2096-9694.2023125

[2] Yan Y, Deng C, Li L, Zhu L, Ye B. Survey of image semantic segmentation methods in the deep learning era. J Image Graphics. 2023;28(11):3342–62. doi: 10.11834/jig.220292

[3] Yanning L, Guobao Z. Design and research on pavement crack segmentation based on convolutional neural network. J Appl Optics. 2024;45(2):373–84. doi: 10.5768/jao202445.0202004

[4] Shui YH, Zhang H, Chen B, Xiong JS, Fu MQ, et al. Lightweight crack segmentation method based on convolutional neural networks. J Hydropower Eng. 2023;42(8):110–20. doi: 10.11660/slfdxb.20230812

[5] Amjoud AB, Amrouch M. Object detection using deep learning, cnns and vision transformers: a review. IEEE Access. 2023;11:35479–516. doi: 10.1109/access.2023.3266093

[6] Long J, Shelhamer E, Darrell T. Fully convolutional networks for semantic segmentation. In: Proceedings of the IEEE Conference on Computer Vision and Pattern Recognition (CVPR). 2015. pp. 3431–40. doi: 10.1109/CVPR.2015.7298965 27244717

[7] Shu JP, Li J, Ma HB, Duan YF, Zhao WJ. Crack detection in ultra-large images based on feature pyramid network. J Civ Environ Eng (Chin English). 2022;44(3):8. doi: 10.11835/j.issn.2096-6717.2021.147

[8] Ronneberger O, Fischer P, Brox T. U-Net: convolutional networks for biomedical image segmentation. In: Medical Image Computing and Computer-Assisted Intervention – MICCAI. Cham: Springer; 2015. pp.234–41. doi: 10.1007/978-3-319-24574-4_28

[9] Liu Z, Cao Y, Wang Y, Wang W. Computer vision-based concrete crack detection using U-net fully convolutional networks. Autom Constr. 2019;104:129–39. doi: 10.1016/j.autcon.2019.04.005

[10] Fan L, Zhao H, Li Y, Li S, Zhou R, Chu W. RAO‐UNet: a residual attention and octave UNet for road crack detection via balance loss. IET Intelligent Trans Sys. 2021;16(3):332–43. doi: 10.1049/itr2.12146

[11] Liu F, Wang J, Chen Z, Xu F. Parallel attention based UNet for crack detection. J Comput Res Dev. 2021;58(8):1718–26. doi: 10.7544/issn1000-1239.2021.20210335

[12] Ruan J, Li J, Xiang S. VM-UNet: Vision Mamba UNet for medical image segmentation. arXiv preprint. 2024. doi: 10.48550/arXiv.2402.02491

[13] Wu R, Liu Y, Liang P, Chang Q. H-vmunet: High-order Vision Mamba UNet for medical image segmentation. Neurocomputing. 2025;624:129447. doi: 10.1016/j.neucom.2025.129447

[14] Liu S, Ren YC, Zheng ZX, Niu ZY. UAV image-based building façade crack detection using an improved U-Net. J Civ Environ Eng. 2024;46(1). doi: 10.11835/j.issn.2096-6717.2022.145

[15] Badrinarayanan V, Kendall A, Cipolla R. SegNet: A Deep Convolutional Encoder-Decoder Architecture for Image Segmentation. IEEE Trans Pattern Anal Mach Intell. 2017;39(12):2481–95. doi: 10.1109/TPAMI.2016.2644615 28060704

[16] Zhao H, Shi J, Qi X, Wang X, Jia J. Pyramid scene parsing network. In: Proceedings of the IEEE Conference on Computer Vision and Pattern Recognition (CVPR). 2017. pp. 2881–90.

[17] Li LF, Wang N, Wu B, Zhang X. Improved PSPNet-based bridge crack image segmentation algorithm. Laser Optoelectron Prog. 2021;58(22):2210001. doi: 10.3788/LOP202158.2210001

[18] Wang S, Li L, Zhuang X. AttU-Net: attention U-Net for brain tumor segmentation. In: International MICCAI Brainlesion Workshop. Cham: Springer International Publishing; 2021. pp. 302–11. doi: 10.1007/978-3-031-09002-8_27

[19] Li R, Zheng S, Zhang C, Duan C, Wang L, Atkinson PM. ABCNet: Attentive bilateral contextual network for efficient semantic segmentation of Fine-Resolution remotely sensed imagery. ISPRS J Photogramm Remote Sensing. 2021;181:84–98. doi: 10.1016/j.isprsjprs.2021.09.005

[20] Tang FH, Ding JR, Quan Q, Wang LT, Ning CP, Zhou SK. CMUNext: An efficient medical image segmentation network based on large kernel and skip fusion. In: 2024 IEEE International Symposium on Biomedical Imaging (ISBI). IEEE; 2024. pp. 1–5. doi: 10.1109/ISBI56570.2024.10635609

[21] Paszke A, Chaurasia A, Kim S, Culurciello E. ENet: A deep neural network architecture for real-time semantic segmentation. arXiv preprint arXiv:1606.02147. 2016. doi: 10.48550/arXiv.1606.02147

[22] Li R, Wang L, Zhang C, Duan C, Zheng S. A2-FPN for semantic segmentation of fine-resolution remotely sensed images. Int J Remote Sens. 2022;43(3):1131–55. doi: 10.1080/01431161.2022.2030071

[23] Chen LC, Papandreou G, Kokkinos I, Murphy K, Yuille AL. Semantic image segmentation with deep convolutional nets and fully connected CRFs. arXiv preprint arXiv:1412.7062. 2014. doi: 10.48550/arXiv.1412.7062 28463186

[24] Chen LC, Zhu YK, Papandreou G, Schroff F, Adam H. Encoder-decoder with atrous separable convolution for semantic image segmentation. In: Proceedings of the European Conference on Computer Vision (ECCV). 2018. pp. 801–18.

[25] Xia XH, Su JG, Wang YY, Liu Y, Li MZ. Lightweight pavement crack detection model based on DeepLabv3+. Laser Optoelectron Prog. 2024;61(08):182–91. doi: 10.3788/LOP231323

[26] Tan GJ, Ou J, Ai YM, Yang RC. Bridge crack image segmentation method based on improved DeepLabv3 model. J Jilin Univ (Eng Technol Ed). 2024;54(1):173–9. doi: 10.13229/j.cnki.jdxbgxb.20220205

[27] Zhang B, Zhang Z, Zhang Y. Improved HRNet applied to segmentation and detection of pavement cracks. Bull Survey Mapping. 2022;(3):83. doi: 10.13474/j.cnki.11-2246.2022.0082

[28] Yuan F, Zhang Z, Fang Z. An effective CNN and transformer complementary network for medical image segmentation. Pattern Recogn. 2023;136:109228. doi: 10.1016/j.patcog.2022.109228

[29] Liao ZH, Zhang YC, Yang B, Lin MC, Sun WB, Gao Z. Monocular height estimation of remote sensing images based on Swin Transformer-CNN and its application in highway construction scenarios. Acta Geodaetica et Cartographica Sinica. 2024;53(2):344. doi: 10.11947/j.AGCS.2024.20220607

[30] Chen P, Li P, Wang B, Ding X, Zhang Y, Zhang T, et al. GFSegNet: A multi-scale segmentation model for mining area ground fissures. Int J Appl Earth Obs Geoinf. 2024;128:103788. doi: 10.1016/j.jag.2024.103788

[31] Xie E, Wang W, Yu Z, Anandkumar A, Alvarez JM, Luo P. SegFormer: Simple and efficient design for semantic segmentation with transformers. Adv Neural Inf Process Syst. 2021;34:12077–90. doi: 10.48550/arXiv.2105.15203

[32] Cao H, Wang YY, Chen J, Jiang DS, Zhang XP, Tian Q, et al. Swin-unet: Unet-like pure transformer for medical image segmentation. In: European Conference on Computer Vision. Cham: Springer Nature Switzerland; 2022. pp. 205–18.doi: 10.1007/978-3-031-25066-8_9

[33] Ruan J, Xiang S, Xie M, Liu T, Fu Y. MALUNet: A Multi-Attention and Light-weight UNet for Skin Lesion Segmentation. In: 2022 IEEE International Conference on Bioinformatics and Biomedicine (BIBM). 2022. pp. 1150–6. doi: 10.1109/bibm55620.2022.9995040

[34] Hou Q, Zhang L, Cheng M-M, Feng J. Strip Pooling: Rethinking Spatial Pooling for Scene Parsing. In: 2020 IEEE/CVF Conference on Computer Vision and Pattern Recognition (CVPR). 2020. doi: 10.1109/cvpr42600.2020.00406

[35] Jin H, Wu Y. Boosting deep ensembles with learning rate tuning. arXiv. 2024. doi: 10.48550/arXiv.2410.07564

[36] Zhou P, Xie X, Lin Z, Yan S. Towards understanding convergence and generalization of AdamW. IEEE Trans Pattern Anal Mach Intell. 2024;46(9):6486–93. doi: 10.1109/TPAMI.2024.3382294 38536692

[37] Dais D, Bal İE, Smyrou E, Sarhosis V. Automatic crack classification and segmentation on masonry surfaces using convolutional neural networks and transfer learning. Autom Constr. 2021;125:103606. doi: 10.1016/j.autcon.2021.103606

